# Pediatric splenectomy for hematologic disorders: two-decade experience and prophylactic cholecystectomy outcomes

**DOI:** 10.1186/s12893-025-03107-0

**Published:** 2025-08-09

**Authors:** Oguzhan Uzaslan, Ali Ekber Hakalmaz, Suheyla Ocak, Osman Faruk Senyuz, Senol Emre

**Affiliations:** 1 Department of Pediatric Surgery, Cerrahpaşa Faculty of Medicine, Istanbul University- Cerrahpaşa, Yesilkoy, Istanbul, Turkey; 2 Division of Pediatric Hematology and Oncology, Department of Pediatrics, Cerrahpaşa Faculty of Medicine, Istanbul University-Cerrahpaşa, Istanbul, Turkey

**Keywords:** Hematologic disorders, Splenectomy, Cholecystectomy, Hereditary spherocytosis, Prophylactic cholecystectomy, Hyperbilirubinemia

## Abstract

**Background:**

While splenectomy remains a cornerstone treatment for certain hematologic diseases, controversy persists regarding the optimal timing and indications for prophylactic cholecystectomy. This study evaluates long-term outcomes from a large single-center series.

**Methods:**

We retrospectively analyzed 87 patients (48 male, 39 female) with hematologic disorders who underwent splenectomy between 2003 and 2023. Primary outcomes included improvement in hematologic parameters, resolution of bilirubinemia, prevalence of cholelithiasis, and complication rates. Subgroup analyses examined disease-specific outcomes and age-stratified results.

**Results:**

Hereditary spherocytosis was the predominant diagnosis (70.1%), followed by thalassemia (11.5%) and immune thrombocytopenia (ITP) (10.3%). The mean age at splenectomy was 9.6 years (range 3–17), with a mean interval from diagnosis to surgery of 4.3 years. Spleen size significantly differed across diseases, being largest in rare hematologic disorders (178 mm) and smallest in ITP (97.56 mm). Cholelithiasis was present in 65.6% of hereditary spherocytosis cases and 50% of thalassemia cases but was absent in ITP. Simultaneous cholecystectomy was performed in 55 patients (63.2%), including 10 prophylactic procedures. Postoperatively, hemoglobin levels increased by factors of 1.24, 1.13, and 1.25 in hereditary spherocytosis, thalassemia, and ITP, respectively. Platelet counts increased by factors of 2.24, 2.8, and 6.5, respectively. Bilirubin levels decreased significantly in all groups. Notably, preoperative total bilirubin was significantly higher (*p* = 0.016) in patients selected for prophylactic cholecystectomy (mean 4.17 mg/dL) compared to those without cholecystectomy (mean 1.92 mg/dL).

**Conclusions:**

Splenectomy offers durable improvements in hematologic parameters across various hematologic diseases, with minimal complications. Prophylactic cholecystectomy appears justified in patients with marked hyperbilirubinemia, even without cholelithiasis, particularly in hereditary spherocytosis. The absence of post-splenectomy sepsis in this series supports the continued role of total splenectomy when appropriate vaccination protocols and antibiotic prophylaxis are implemented.

## Background

Hematologic diseases represent a diverse group of disorders characterized by the accelerated destruction of red blood cells, often leading to anemia, jaundice, and the formation of gallstones. When medical management fails to control symptoms or prevent complications, surgical intervention becomes necessary. Splenectomy has long been the definitive treatment for reducing hemolysis, decreasing transfusion requirements, and alleviating symptoms in these patients [1, 2]. The understanding and clinical approach to splenectomy have significantly evolved over the past two decades. A growing understanding of the immunological consequences, particularly the risk of overwhelming post-splenectomy infection (OPSI), has led to more conservative approaches, including partial splenectomy and postponement of surgery until after age five whenever possible, guided by specific risk assessments and vaccination strategies [2–4]. However, controversy remains regarding indications for total versus partial splenectomy, particularly balancing long-term efficacy with preservation of immune function [5], and the role of simultaneous cholecystectomy, particularly in the absence of gallstones [6]. A key clinical question is whether the benefits of prophylactic cholecystectomy performed concurrently with splenectomy outweigh the additional surgical risks. Some national guidelines (e.g., the Turkish Society of Hematology guidelines [7]) do not support the use of prophylactic cholecystectomy in hereditary spherocytosis in the absence of gallstones. This recommendation persists despite reports indicating high rates of post-splenectomy cholelithiasis in this patient population [8]. Nevertheless, clinical practice varies widely, with some centers routinely performing prophylactic cholecystectomy due to high rates of gallstone formation after splenectomy, particularly in patients with marked hyperbilirubinemia [6, 9]. This study specifically investigates whether elevated preoperative bilirubin levels can serve as a practical indicator for identifying patients at higher risk who might benefit most from prophylactic cholecystectomy, aiming to provide data to inform this ongoing debate.

This study presents a comprehensive analysis of 87 pediatric patients who underwent splenectomy for hematologic diseases at our center over a 20-year period (2003–2023). While immune thrombocytopenia (ITP) is not classified as a hemolytic disorder but rather as an autoimmune condition affecting platelet destruction, it was included in this study to provide comprehensive insights into splenectomy outcomes across various benign hematologic and immunologic disorders requiring splenectomy. The inclusion of ITP patients allows for comparative analysis of surgical outcomes and complications across different pathophysiological mechanisms, thereby enhancing the generalizability of our findings to the broader pediatric population undergoing splenectomy. By analyzing disease-specific outcomes, age-related differences, and the effects of prophylactic cholecystectomy, we aim to provide valuable data to guide clinical decision-making in this complex patient group. Our primary objectives are to: (a) evaluate the long-term hematological outcomes following splenectomy across different hematologic diseases; (b) assess the role of prophylactic cholecystectomy in patients without gallstones but with elevated bilirubin levels; and (c) examine the safety profile of these procedures in the pediatric population with extended follow-up.

## Methods

This retrospective study was approved by the Istanbul University-Cerrahpaşa Clinical Research Ethics Committee (Approval Number: E-83045809-804.01-968615). We analyzed all pediatric patients (age 0–18 years) who underwent splenectomy for hematologic diseases at our Pediatric Surgery Clinic between 2003 and 2023.

Data Collection We extracted information from operating room logs, patient records, archive files, and the hospital’s electronic medical record system. Data collected included:

- ***Demographics***: gender, birth date, age at diagnosis, age at operation.
- ***Clinical characteristics***: primary diagnosis, spleen size (longitudinal axis measurement), presence of splenomegaly, accessory spleen detection, presence of cholelithiasis.
- ***Laboratory parameters***: preoperative and postoperative (1 month and 1 year) complete blood count (hemoglobin, leukocyte, platelet counts) and biochemical values (total and conjugated bilirubin).
- ***Surgical details***: splenectomy technique (open vs. laparoscopic), simultaneous or subsequent cholecystectomy.
- ***Perioperative management***: vaccination status, prophylactic antibiotic use, antiplatelet medication use.
- ***Postoperative complications***: wound complications, bleeding, fluid collections, infections, sepsis, vascular thrombosis, and pulmonary hypertension.

### Splenectomy indications and patient selection

All patients were analyzed according to their primary hematologic diagnoses. Splenectomy indications were determined by pediatric hematologists based on established criteria and international guidelines:

- Hereditary spherocytosis: Transfusion dependency, growth retardation, severe anemia, or frequent hemolytic crises.
- Thalassemia: Transfusion dependency with secondary hypersplenism and iron overload complications.
- ITP: Refractory chronic disease unresponsive to medical therapy.
- Other conditions: Case-by-case multidisciplinary evaluation considering disease severity and treatment response.

ITP patients were analyzed as a distinct subgroup. Although ITP does not involve hemolysis, it was included to provide comprehensive insights into splenectomy outcomes across various hematologic and immunologic disorders. Secondary anemia observed in ITP patients (mean Hb: 10.7 g/dL) was attributed to chronic bleeding episodes secondary to severe thrombocytopenia, while anemia in hematologic disorders reflected the underlying disease pathophysiology.

### Surgical protocol

Before 2017, splenectomy was performed using open techniques; thereafter, reflecting evolving institutional practice and surgeon preference, laparoscopic approaches were adopted. Preoperative vaccination protocols included pneumococcal, meningococcal, and *Haemophilus influenzae* type B vaccines, administered at least two weeks before surgery whenever possible. Postoperative antibiotic prophylaxis consisted of ampicillin-sulbactam followed by intramuscular depot penicillin. Antiplatelet therapy was initiated for patients with thrombocytosis exceeding 750 × 10⁹/L before 2015 and 1000 × 10⁹/L thereafter based on evolving evidence regarding thrombotic risk stratification [10].

### Follow-up protocol

 Patients were followed regularly in our pediatric surgery and hematology clinics. Post-splenectomy surveillance included clinical evaluation at 1, 6, and 12 months, then annually. Parents were educated about OPSI symptoms and instructed to seek immediate medical attention for fever > 38 °C. Any hospitalization for infection was documented and reviewed to assess for potential OPSI.

### Statistical analysis

Data were analyzed using GraphPad Prism v.10.0.0. Normality was assessed using the Shapiro-Wilk test. One-way ANOVA or Mann-Whitney U tests were used for intergroup comparisons based on data distribution. The Paired Sample’s t-test was used for intragroup comparisons. Proportions were evaluated using the Chi-square or Fisher’s exact test, with significance set at *p* < 0.05.

## Results

### Patient demographics and disease distribution

**Fig. 1:**
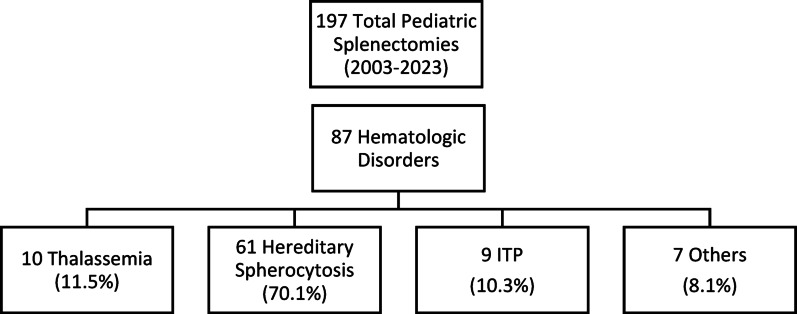
Patient selection flowchart showing distribution of 87 pediatric patients who underwent splenectomy for hematologic disorders over 20 years (2003–2023)

Between 2003 and 2023, 197 splenectomies were performed at our clinic; 87 of these were indicated for hematologic diseases. Among these 87 patients, 48 were male (55.2%) and 39 were female (44.8%). The mean age at diagnosis was 5.3 years (range 0–16 years), while the mean age at splenectomy was 9.6 years (range 3–17 years). The mean interval from diagnosis to splenectomy was 4.3 years (range 0–17 years). The primary indications for splenectomy included hereditary spherocytosis in 61 patients (70.1%), thalassemia in 10 (11.5%), immune thrombocytopenic purpura (ITP) in 9 (10.3%), sickle cell anemia in 2, congenital dyserythropoietic anemia in 2, hemophagocytic lymphohistiocytosis in 1 (indicated for refractory massive splenomegaly with persistent hypersplenism unresponsive to standard medical therapy), congenital erythropoietic porphyria in 1, and undiagnosed hemolytic anemia in 1 patient. (Fig. 1)

Seventy-two splenectomies (82.8%) were performed via laparotomy, and 15 (17.2%) were performed laparoscopically. Simultaneous cholecystectomy was performed in 55 cases (63.2%), with 10 of these being prophylactic (performed in the absence of gallstones). One patient developed symptomatic cholelithiasis after splenectomy, necessitating a later laparoscopic cholecystectomy. Additionally, one patient had undergone cholecystectomy prior to splenectomy. Analysis of demographic and clinical characteristics across disease groups showed no significant differences regarding gender, age at diagnosis, age at surgery, or the interval between diagnosis and surgery. However, the mean spleen size was notably larger in the “other diseases” group (178 mm) compared to thalassemia (159.3 mm), hereditary spherocytosis (135.7 mm), and ITP (97.56 mm), with these differences being statistically significant. None of the 9 ITP patients had cholelithiasis, and none underwent prophylactic cholecystectomy, marking a statistically significant contrast with the other disease groups (Table 1).

**Table 1:** Demographic data and clinical findings according to disease groups

Variables	Hereditary Spherocytosis (*n* = 61)	Thalassemia (*n* = 10)	ITP (*n* = 9)	Other (*n* = 7)	*p*
M/F	30/31	6/4	7/2	5/2	0.157
Age at diagnosis (years)	5.66 ± 5.67	4.62 ± 3.11	6.14 ± 4.77	3.00 ± 3.05	0.168
Age at operation (years)	9,148 ± 4,183	11.00 ± 3.464	10.56 ± 5.003	9,714 ± 3,684	0.189
Time from diagnosis to surgery (years)	3,943 ± 3,749	6,375 ± 6,523	4,286 ± 2,430	6,714 ± 4,386	0.076
Spleen size (mm)	135.7 ± 31.23	159.3 ± 34.65	97.56 ± 11.31	178.0 ± 58.27	< 0.001*
Accessory spleen n (%)	20 (32.78%)	2 (20.0%)	1 (11.1%)	1 (14.3%)	0.261
The presence of cholelithiasis	40 (65.57%)	5 (50.0%)	0	2 (28.6%)	< 0.001**
Prophylactic Cholecystectomy	7 (11,5%)	2(20%)	0	1(14,3%)	
Cholecystectomy	47 (77.05%)	6 (60.0%)	0	4 (57.14%)	< 0.005***

Bold values indicate statistically significant differences (*p* < 0.05)

* No significant difference was observed between the Thalassemia and Other diseases groups regarding spleen size (*p* = 0.418). All other group comparisons showed statistically significant differences for this parameter

** Cholelithiasis rate was significantly lower in the ITP group compared to the Hereditary Spherocytosis and Thalassemia groups

*** The cholecystectomy rate was significantly lower in the ITP group compared to all other groups

### Hematological outcomes

Regarding CBC parameters, patients with thalassemia had significantly lower preoperative and postoperative hemoglobin levels compared to those with hereditary spherocytosis and ITP. Preoperative platelet levels were significantly lower in ITP cases than in the other groups. A notable increase in both hemoglobin and platelet levels was observed across all disease groups when comparing preoperative values to postoperative 1-year values. Postoperative 1-year hemoglobin levels increased by a factor of 1.24 in hereditary spherocytosis, 1.13 in thalassemia, and 1.25 in ITP compared to preoperative levels. Similarly, postoperative 1-year platelet levels rose by factors of 2.24, 2.8, and 6.5, respectively (Table 2).

**Table 2:** Selected hematological outcomes across disease groups

Parameter	Hereditary Spherocytosis (*n* = 61)	Thalassemia (*n* = 10)	ITP (*n* = 9)	Other Hematologic Diseases (*n* = 7)	*p*-value
Hemoglobin (g/dL)					
• Preoperative	10.03 ± 1.52	8.14 ± 0.86*	10.74 ± 2.13	10.24 ± 2.34	**0.004**
• Postop 1-month	12.15 ± 1.18	9.67 ± 1.19*	11.87 ± 0.93	11.24 ± 2.23	**< 0.001**
• Postop 1-year	12.44 ± 1.15	9.21 ± 1.46*	13.44 ± 1.46	12.27 ± 1.23	**< 0.001**
• Change factor†	1.24	1.13	1.25	1.20	**-**
Leukocyte (×10⁹/L)					
• Preoperative	8.2 ± 2.9	8.65 ± 5.73	7.66 ± 2.24	6.64 ± 2.63	0.619
• Postop 1-month	10.62 ± 4.12	12.4 ± 4.36	13.25 ± 4.39	9.91 ± 3.02	0.240
• Postop 1-year	8.98 ± 2.58	9.91 ± 3.02	10.64 ± 5.26	9.29 ± 4.47	0.622
• Change factor†	1.09	1.15	1.39	1.40	-
Platelet (×10⁹/L)					
• Preoperative	279.7 ± 116.5	258.2 ± 157.1	39.56 ± 25.23*†	227.9 ± 120.5	**< 0.001**
• Postop 1-month	729.5 ± 267.5	889.8 ± 274.3*	407.6 ± 193.1†	620.6 ± 384.9	**0.003**
• Postop 1-year	627.6 ± 222.3	723.8 ± 207.9	256.7 ± 76.8*†	504.9 ± 173.4	**< 0.001**
• Change factor†	2.24	2.80	6.50	2.22	**-**

Significantly different from Hereditary Spherocytosis (*p* < 0.05) †: Significantly different from all other groups (*p* < 0.05) ‡: Postoperative 1-year value divided by preoperative value Bold values indicate statistically significant differences (*p* < 0.05). Bold values indicate statistically significant differences (*p* < 0.05)

### Age-related findings and prophylactic cholecystectomy

**Fig. 2:**
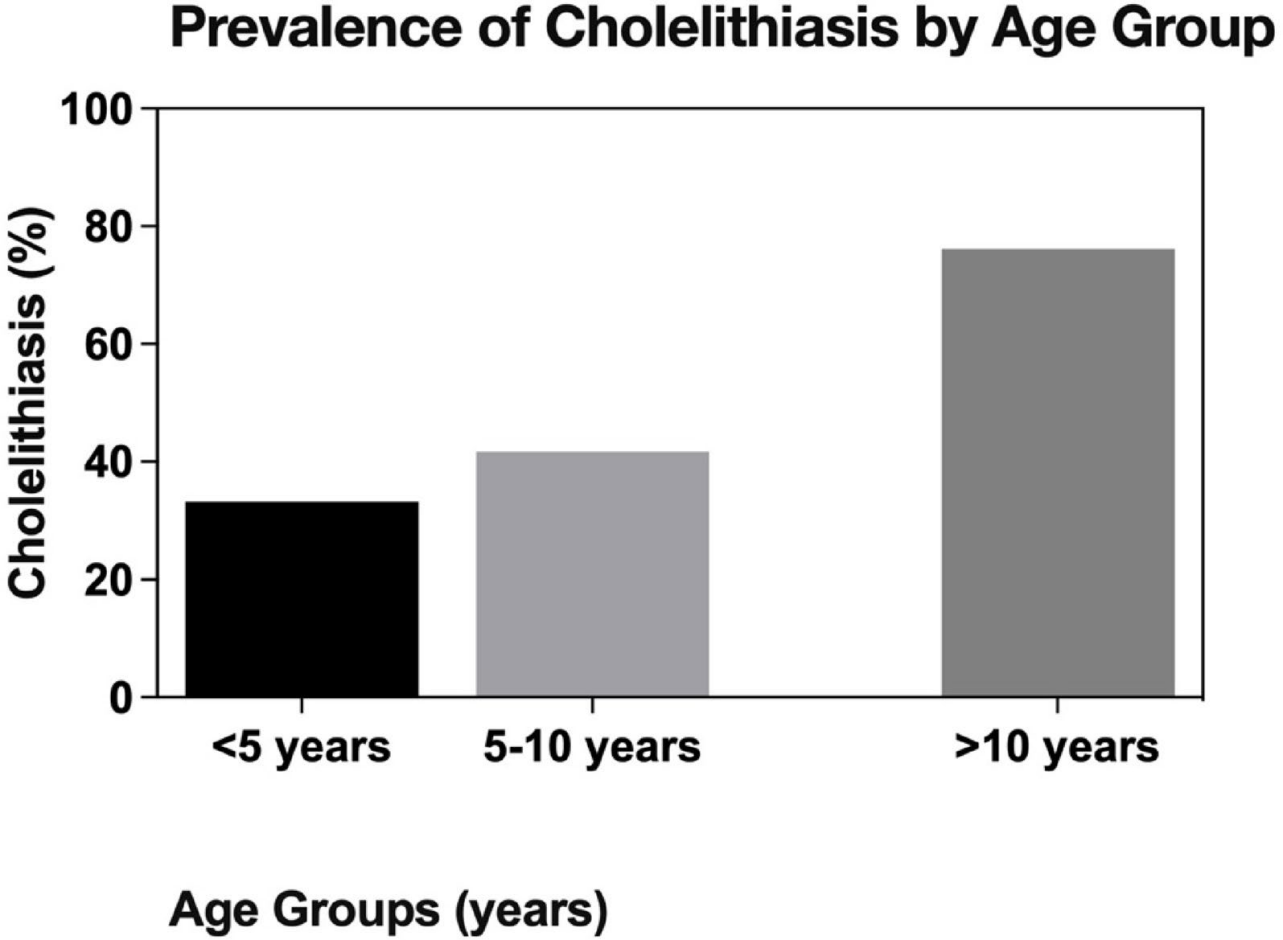
Prevalence of cholelithiasis by age group in pediatric patients undergoing splenectomy. The prevalence increased significantly with age (*p* = 0.002), demonstrating higher gallstone formation risk in older children

Age-stratified analysis revealed significant differences in spleen size, measuring 112.3 mm, 133.6 mm, and 145.5 mm for patients aged < 5, 5–10, and > 10 years, respectively (*p* = 0.022). The prevalence of cholelithiasis increased significantly with age (33.3%, 41.7%, and 76.2% in the respective age groups; *p* = 0.002). Prophylactic cholecystectomy was not performed in any patient under 5 years old but was performed in 11.1% of patients aged 5–10 years and 14.3% of those over 10 years (Fig. 2). Notably, the postoperative leukocyte response differed significantly by age; children under 5 years showed markedly elevated levels (mean 19.18 × 10⁹/L) compared to older children (mean 10.44–10.6 × 10⁹/L) at one month post-splenectomy (*p* < 0.001).

### Bilirubin levels and prophylactic cholecystectomy

**Fig. 3:**
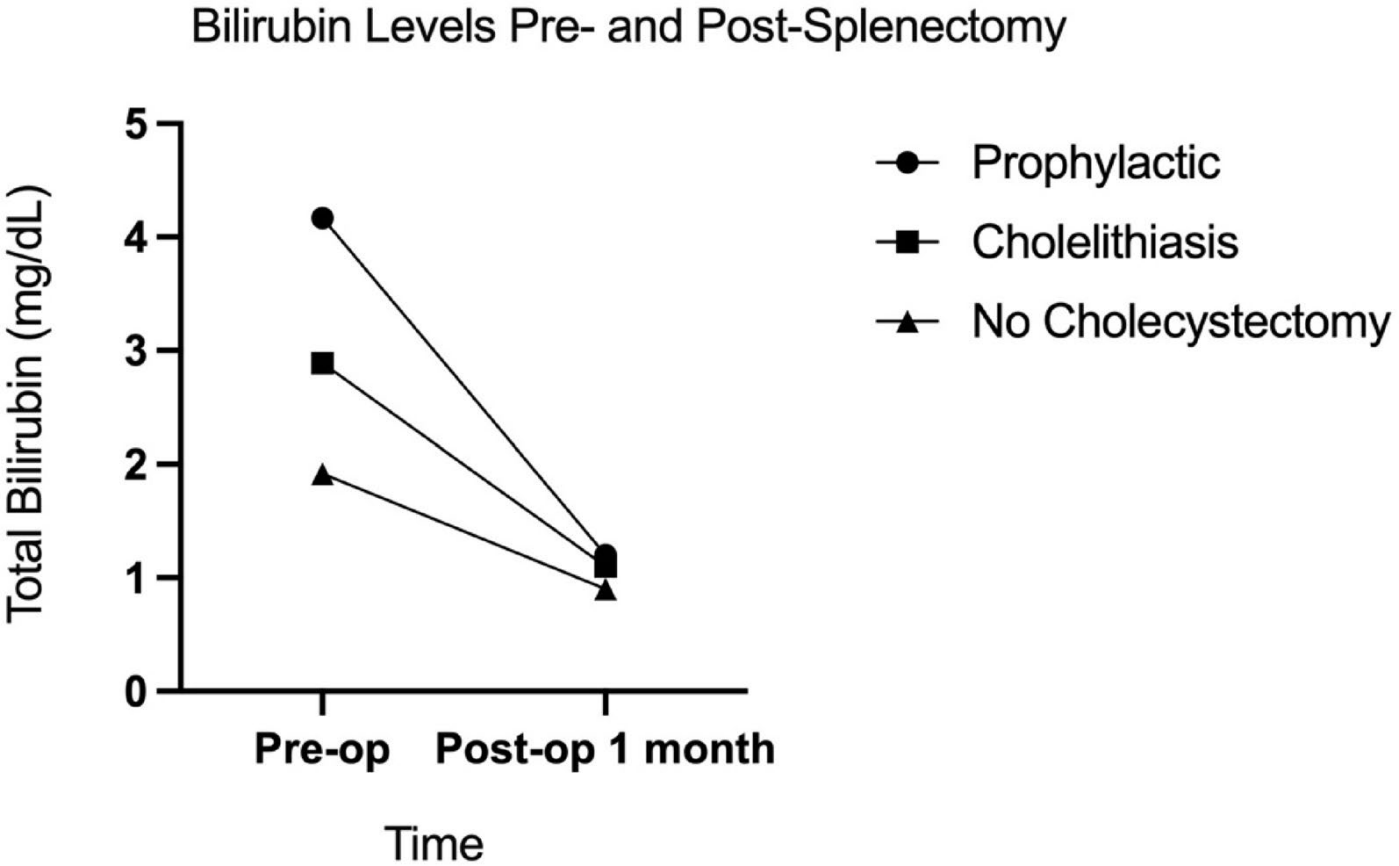
Changes in total bilirubin levels from preoperative to 1-month postoperative period across different cholecystectomy groups. Patients selected for prophylactic cholecystectomy had significantly higher preoperative bilirubin levels (*p* = 0.016), which normalized postoperatively across all groups

Analysis of bilirubin levels according to cholelithiasis and cholecystectomy status revealed important patterns. Preoperative total bilirubin levels were significantly higher in patients who underwent prophylactic cholecystectomy (mean 4.17 mg/dL) compared to those who did not undergo cholecystectomy (mean 1.92 mg/dL) (*p* = 0.016). Similarly, preoperative conjugated bilirubin levels were significantly higher in the prophylactic cholecystectomy group (mean 1.77 mg/dL) compared to the non-cholecystectomy group (mean 0.68 mg/dL) (*p* = 0.045). Total and conjugated bilirubin levels decreased significantly one month after splenectomy across all groups (Fig. 3), with no significant differences noted between the groups in postoperative values (Table 3).

**Table 3:** Bilirubin levels according to cholelithiasis and cholecystectomy history

Parameter	No Cholecystectomy (*n* = 31)	Cholecystectomy for Cholelithiasis (*n* = 4)	Prophylactic Cholecystectomy (*n* = 10)	p-value
*Total Bilirubin (mg/dL)*				
Preoperative	1.92 ± 1.57*	3.00 ± 2.04	4.17 ± 4.81†	**0.016**
Postop 1-month	0.89 ± 0.61	0.86 ± 0.54	1.04 ± 0.54	0.666
p-value (pre vs. 1-month)	**< 0.001**	**< 0.001**	**0.018**	
*Conjugated Bilirubin (mg/dL)*				
Preoperative	0.68 ± 0.75*	0.92 ± 1.22	1.77 ± 2.53†	**0.045**
Postop 1-month	0.25 ± 0.16	0.27 ± 0.21	0.29 ± 0.19	0.808
p-value (pre vs. 1-month)	**< 0.001**	**< 0.001**	**0.034**	

*: Significantly lower than prophylactic cholecystectomy group (*p* < 0.05) †: Significantly higher than no cholecystectomy group (*p* < 0.05) Bold values indicate statistically significant differences (*p* < 0.05). Bold values indicate statistically significant differences (*p* < 0.05)

#### Surgical technique outcomes

Laparoscopic approach (*n* = 15) was associated with shorter operative time (mean 145 vs. 168 min, *p* = 0.032) and reduced hospital stay (mean 3.2 vs. 4.7 days, *p* = 0.018) compared to open approach (*n* = 72). Complication rates were comparable between techniques.

### Complications

Complications were minimal across all disease groups during the mean follow-up period of 12.04 years (range 1–21 years). Minor complications included superficial wound infections (*n* = 2), a hematoma at the splenectomy site (*n* = 1), and a fluid collection at the cholecystectomy site (*n* = 1). Two patients required hospitalization for respiratory infections unrelated to the surgery. Importantly, no patient developed post-splenectomy sepsis (PSS) during the entire follow-up period. Two significant late adverse events occurred: one patient with hemophagocytic lymphohistiocytosis died 1.5 years post-splenectomy due to intestinal perforation unrelated to the initial procedure, and one patient with alpha thalassemia developed intrahepatic bile duct stones and recurrent cholangitis 9 years post-surgery, ultimately requiring liver transplantation.

## Discussion

Our 20-year experience with splenectomy for hematologic diseases demonstrates excellent long-term outcomes with minimal complications. The predominance of hereditary spherocytosis in our series (70.1%) [11] reflects the common indications for splenectomy in our region, differing from the epidemiology in other geographic areas where thalassemia [12] or sickle cell disease [13] may be more prevalent causes requiring such intervention, highlighting the influence of regional epidemiology on hematologic disease management [11–13]. The mean age at splenectomy in our cohort (9.6 years) reflects the recommended practice of postponing surgery until after age 5 whenever feasible to minimize the risk of OPSI [3, 4]. This approach aligns with current guidelines recognizing the critical immunological role of the spleen in early childhood [3, 4]. Even in severely affected patients, we found that delaying splenectomy did not compromise long-term outcomes, supporting conservative timing when clinically appropriate.

The decision for prophylactic cholecystectomy in hereditary spherocytosis patients without evident gallstones was based on persistently elevated bilirubin levels post-splenectomy, suggesting potential co-inheritance of Gilbert syndrome or residual biliary risk factors. While splenectomy effectively halts hemolysis, concurrent cholecystectomy eliminates future gallstone-related complications and avoids additional surgical procedures in these pediatric patients [3, 14–16]. However, we acknowledge that genetic confirmation of Gilbert syndrome was not routinely performed, and long-term prospective studies are needed to validate this approach.

Despite increasing interest in spleen-preserving approaches, our series supports the continued validity of total splenectomy when performed with appropriate immunoprophylaxis. Importantly, no cases of OPSI were observed during a mean follow-up of 12 years. This suggests that the infectious risks traditionally associated with total splenectomy can be effectively mitigated in the contemporary era through current vaccination and antibiotic protocols [17], reinforcing the safety of this approach in our experience. While partial splenectomy has been advocated as a strategy to preserve splenic immune function, its long-term efficacy remains controversial. Several studies and systematic reviews have reported variable rates of splenic remnant regrowth and recurrence of hemolysis or cytopenias, with some patients eventually requiring completion splenectomy due to recurrent hemolysis or symptomatic splenomegaly [5, 18]. Considering the technical complexity and potential long-term failure of partial splenectomy, our findings support total splenectomy as a reliable and definitive treatment option for selected pediatric patients with hematologic disorders.

A notable secondary finding was the significantly elevated postoperative leukocyte count observed one month after surgery in children younger than five years. While the clinical significance of this transient elevation requires further investigation, it might reflect a more robust, age-related inflammatory or immunological response to surgery in younger children.

Ensuring complete removal of splenic tissue is crucial for therapeutic success, particularly in immune thrombocytopenia (ITP), where splenectomy remains an important second-line treatment option [19]. The 28.7% prevalence of accessory spleens observed in our cohort aligns with previously reported rates, which vary widely but are often cited between 10% and 30% based on surgical and autopsy series [20, 21]. This finding underscores the importance of meticulous intraoperative exploration, particularly in ITP, where the persistence of functional splenic tissue from an accessory spleen is a well-recognized cause of surgical failure or early relapse, potentially necessitating reoperation [22]. The presence of accessory spleens did not significantly affect complication rates (4.0% vs. 3.2% in patients without accessory spleens, *p* = 0.84) or surgical outcomes. Our routine use of preoperative radionuclide scintigraphy, particularly for ITP cases, may have contributed to the favorable surgical response in our cohort by facilitating the detection of ectopic splenic tissue before resection. Integrating functional imaging techniques with thorough intraoperative assessment remains a key strategy to maximize the therapeutic efficacy of splenectomy in these patients [23].

## Conclusions

This 20-year experience supports the continued role of splenectomy as a safe and effective treatment for pediatric hematologic diseases, providing durable hematological benefits. Our findings suggest that prophylactic cholecystectomy during splenectomy may be justified in patients with significant hyperbilirubinemia, even without cholelithiasis, particularly in those with hereditary spherocytosis. The absence of PSS in our cohort demonstrates that with proper vaccination and antibiotic prophylaxis, the infectious risks of total splenectomy can be effectively managed in the contemporary era. Future prospective multicenter studies are needed to establish definitive criteria for prophylactic cholecystectomy and validate our bilirubin-based selection approach across different institutions.

## Data Availability

The datasets used and/or analysed during the current study are available from the corresponding author on reasonable request.
